# Nonreference Medical Image Edge Map Measure

**DOI:** 10.1155/2014/931375

**Published:** 2014-07-15

**Authors:** Karen Panetta, Chen Gao, Sos Agaian, Shahan Nercessian

**Affiliations:** ^1^Department of Electrical and Computer Engineering, Tufts University, Medford, MA 02155, USA; ^2^Department of Electrical and Computer Engineering, The University of Texas at San Antonio, San Antonio, TX 78249, USA; ^3^MIT Lincoln Laboratory, Lexington, MA 02421, USA

## Abstract

Edge detection is a key step in medical image processing. It is widely used to extract features, perform segmentation, and further assist in diagnosis. A poor quality edge map can result in false alarms and misses in cancer detection algorithms. Therefore, it is necessary to have a reliable edge measure to assist in selecting the optimal edge map. Existing reference based edge measures require a ground truth edge map to evaluate the similarity between the generated edge map and the ground truth. However, the ground truth images are not available for medical images. Therefore, a nonreference edge measure is ideal for medical image processing applications. In this paper, a nonreference reconstruction based edge map evaluation (NREM) is proposed. The theoretical basis is that a good edge map keeps the structure and details of the original image thus would yield a good reconstructed image. The NREM is based on comparing the similarity between the reconstructed image with the original image using this concept. The edge measure is used for selecting the optimal edge detection algorithm and optimal parameters for the algorithm. Experimental results show that the quantitative evaluations given by the edge measure have good correlations with human visual analysis.

## 1. Introduction 

Edge detection is an essential preprocessing step for early cancer detection and diagnosis in medical image processing such as medical image segmentation, registration, and reconstruction. For example, accurate edge detection algorithms can be used to track the size of a tumor and this information can help to monitor whether the treatment is effective or not. Traditional edge detection algorithms can be grouped into two categories: one utilizes the first order gradient information and the other uses second derivative zero crossing information. Some popular algorithms include Sobel, Robert, Prewitt, Laplacian, LoG, and Canny algorithm [1]. Some other state-of-the-art edge detection algorithms also include Partial Derivatives of Boolean Functions [2] and the Alpha Weighted Quadratic Filter [3]. Although the performance of most of these detectors is acceptable for simple noise free images, the case is dramatically different for medical images subjected to noises from the acquisition systems [4]. Unfortunately, medical images usually suffer from low contrast or poor resolution due to the limitation of hardware systems or the exposure time. Therefore, it is necessary to have a reliable evaluation method to measure the performance of different edge detection algorithms and help select the optimal algorithm for specific medical applications.

Many edge measures have been proposed including the full reference edge measure [5], the nonreference measure [6, 7], and the subjective evaluation. The full reference edge measure requires a ground truth image as a reference and compares the similarity between candidate edge maps and the ground truth edge map. However, the ground truth image is not available for medical images. Subjective evaluation ratings by medical experts are the most widely accepted evaluation method in medical image processing. This approach avoids the use of ground truth edge maps. However, it is impossible to remove all the bias and the results can still be inconsistent. Furthermore, subjective evaluation is expensive with respect to time and resources; thus it is difficult to be automated.

The nonreference based method does not require a ground truth and it can be automated. Unfortunately, the existing nonreference edge measures are still far from ideal. A nonreference based edge map evaluation should only use the information from the resultant edge map and the original image itself to make an evaluation. Yitzhaky and Peli proposed a probabilistic based nonreference measure using the receiver operating characteristics (ROC) [7]. Their method first estimates a ground truth edge map using automatic statistical analysis of the correlation of detection results produced using different detector parameters. Then, the edge map which is most similar to the estimated ground truth is selected as the optimal edge detection results. This method balances specificity and sensitivity. However, this method suffers from its bias regarding the generation of the estimated ground truth, because the candidate edge maps used can directly affect the estimated ground truth. Therefore, if the majority of the edge maps used are not of adequate quality or fail to extract certain features, this will be reflected in the derived estimated ground truth. Also, since the original image data is not used, there is no way to indicate how well the best determined edge detector output from this approach corresponds to the original image.

In this paper, we present a reconstruction based nonreference edge measure. The theoretical basis of this method is that a good edge map captures the essential structures and details of the original images. Therefore, the reconstruction using the pixel information on a better edge map would be more similar to the original image. In our method, the edge measure is composed of two components: the first is the gradient based structural similarity measure between the original image and the reconstructed image, and the second component is the penalty factor. For instance, the reconstructions from edge maps with the most edge pixels have the greatest similarity measure. However, they utilize more information from the original image. To compensate for this, a penalty factor which is inversely proportional to the number of edge pixels is also included in the formulation of the measure. In other words, we want a measure that chooses the optimal edge map as the one that shows the structural details in the image with minimal information and minimal false positives.

The rest of this paper is organized as follows.  Section 2 reviews existing reconstruction methods and the similarity measures. More details of Yitzhaky's edge measure are also reviewed in this section.  Section 3 presents a new nonreference edge measure (NREM).  Section 4 presents the experimental results of using the NREM on choosing the optimal edge detection algorithm and optimal operating parameters for medical images. The comparisons against Yitzhaky's edge measure are also presented in this section. The conclusions are discussed in Section 5.

## 2. Background

The new NREM is a reconstruction based edge measure. In this section, the existing reconstruction methods are reviewed. Similarity measures are used to compare the correlation between the reconstructed image and the original image. Varieties of similarity measures are also reviewed in this section. Lastly, as a comparison to the NREM, theoretical analysis and the basic steps of Yitzhaky's measure are shown in this section.

### 2.1. Reconstruction

Interpolation has been widely used to obtain the missed pixels from the original image. In the context of reconstruction, the pixels along edges are used to predict the pixel values in the smooth areas. One of the linear interpolation methods [8] can be described as follows. For each pixel location (*i*, *j*) ∉ *e*_*D*_, the algorithm searches in the four horizontal and vertical directions and four diagonal directions for the nearest pixel in the given direction that ∈*e*_*D*_. The inverse of the distances of the first pixel encountered in each direction from the given pixel *d*_*k*_ is then used as the weights for the weighted average of their respective image intensity values *t*_*k*_, yielding the reconstructed intensity value for the given pixel. Thus, reconstruction is carried out for each pixel location (*i*, *j*) ∉ *e*_*D*_ by the following:

(1) $$\begin{array}{c} r\left(i,j\right)=\frac{\sum_{k=1}^{8}\left(1/d_{k}\right)t_{k}}{\sum_{k=1}^{8}\left(1/d_{k}\right)}. \end{array}$$

An improvement of (1) is using a weighted median instead of weighted mean to make it more robust to noise. Another modification utilizes the central weighted median. The central weighted median of a sequence *x* with weights *w* is given by (2), where the weights are inversely related to the distance (3) and ⋄ is the replication operator representing the fact that intensity value *t*_*i*_ is repeated *w*_*i*_ times in the sequence of median calculation in the following:

(2) $$\begin{array}{c} r\left(i,j\right)=\text{median}\left(t_{1}⋄w_{1},t_{2}⋄w_{2},\ldots ,t_{8}⋄w_{8}\right), \end{array}$$

(3) $$\begin{array}{c} w_{k}=\text{round}\left(\frac{100}{d_{k}}\right). \end{array}$$

Another type of reconstruction methods is based on the partial differential equation (PDE) discretization proposed by Ballester et al. [9]. In such methods, high order PDEs are designed to restore smooth regions as well as thin structures. These reconstruction based methods have clear advantages for effectively incorporating the original image information on the edge pixels. In nonreference measures this information is essential because no ground truth exists.

### 2.2. Similarity Measures

To compare the similarity between two images, the most commonly adopted methods are the statistical methods including the pixel-wised mean square error (MSE) and mean absolute error (MAE). The MSE and MAE between two images *x* and *y* are defined as shown in (4). In (4), *x* and *y* represent the two images for comparison and *i* and *j* represent the pixel locations. These statistical methods have clear physical meanings and are straightforward. Under these definitions, two images with more similarity have lower MSE or MAE:

(4) $$\begin{array}{c} \text{MSE}=\frac{1}{MN}\overset{M}{\underset{i=1}{\sum }}\overset{N}{\underset{j=1}{\sum }}{\left[x\left(i,j\right)-y\left(i,j\right)\right]}^{2},\\ \text{MAE}=\frac{1}{MN}\overset{M}{\underset{i=1}{\sum }}\overset{N}{\underset{j=1}{\sum }}\left|x\left(i,j\right)-y\left(i,j\right)\right|. \end{array}$$

However, these statistical methods do not take into consideration the human visual system (HVS) properties. Therefore, they are inappropriate to be used as reliable measures for medical images. Bovik's structural similarity measure (SSIM) [10] is based on the hypothesis that human visual system (HVS) is highly adapted for extracting structural information. The SSIM measure defines the similarity of two images as a function of luminance, contrast, and structure, where the luminance, contrast, and structure are defined as

(5) l(x,y)=2μxμy+C1μx2+μy2+C1,c(x,y)=2σxσy+C2σx2+σy2+C2,s(x,y)=σxy+C3σxσy+C3.

Given two images *x* and *y*, the *μ*_*x*_ and *μ*_*y*_ represent the means, *σ*_*x*_ and *σ*_*y*_ represent the standard deviation of the *x* and *y* image, respectively, and *σ*_*xy*_ represent the covariance of *x* and *y*. *C*_1_, *C*_2_, and *C*_3_ represent constant values. SSIM is a combination of luminance, contrast, and structure measure and it is defined as shown in (6). The SSIM is applied on nonoverlapping windows. Thus the mean of the SSIM values over the entire image (MSSIM (7)) is used to indicate the similarity between two images:

(6) $$\begin{array}{c} \text{SSIM}\left(x,y\right)=\left(\frac{2\mu_{x}\mu_{y}+C_{1}}{\mu_{x}^{2}+\mu_{y}^{2}+C_{1}}\right)\left(\frac{2\sigma_{x}\sigma_{y}+C_{2}}{\sigma_{x}^{2}+\sigma_{y}^{2}+C_{2}}\right), \end{array}$$

(7) $$\begin{array}{c} \text{MSSIM}\left(x,y\right)=\frac{1}{N}\overset{N}{\underset{i=1}{\sum }}\text{SSIM}\left(x_{i},y_{i}\right). \end{array}$$

### 2.3. Yitzhaky and Peli's Edge Measure

Yitzhaky and Peli proposed an objective edge detection evaluation method in [7]. In this paper, the new measure is compared with this method. Yitzhaky's edge measure is briefly reviewed in the following section.

Yitzhaky's edge measure [7] is a nonreference edge measure. This method performs statistical analysis of the correspondence of detection results produced using different detector parameters. The statistical measures are used to estimate the ground truth edge map considering the tradeoff between the true and false edges and to extract the best detector's parameter sets. In Yitzhaky's method, first, an estimated ground truth is automatically constructed by examining the corresponding threshold receiver operating characteristics (CT-ROC) curve, given a range of detection results obtained from different detection parameter sets. Then, the single parameter set that provides the most similar edge map to the estimated ground truth edge map is identified. The major steps of Yitzhaky's edge measure are summarized in Table 1.

The original Yitzhaky's edge measure performs a general automatic self-evaluation and parameter selection within a range of examined detector parameters. It assumes that the best detection of a certain edge detector in a given image is that which is most consistent with the variety of detection outputs that can be produced by the detection algorithm when different parameters are used. Therefore, by adopting *N* edge detection results using *N* sets of parameters of one edge detection algorithm, this algorithm can be used to select the optimal operating parameters. If the *N* edge detection results are obtained from *N* edge detectors, this algorithm can be used to compare performances between detection approaches.

## 3. New Nonreference Edge Map Measure

**Figure 1:**
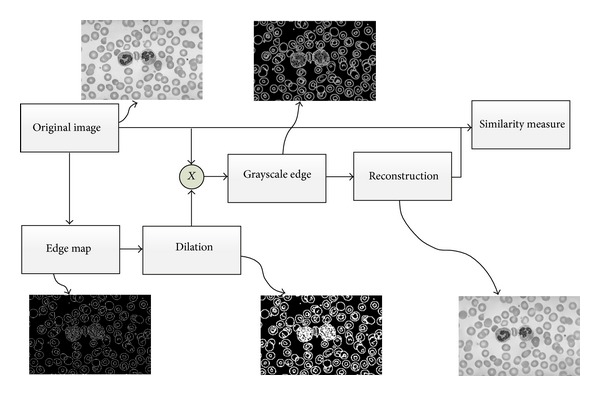
Flow and the intermediate results of the new nonreference edge measure.

A generalized block diagram and intermediate results of the established nonreference reconstruction based edge measure (NREM) are shown in Figure 1. It consists of three major steps: grayscale edge map generation, reconstruction, and similarity measure.

(*1) Grayscale Edge Map Generation.* The edge detection algorithm to be evaluated is applied in this step. Regular edge detection algorithms such as Canny, Sobel, Log, and Roberts can be used. Furthermore, some state-of-the-art edge detection algorithms designed specifically for the medical image applications such as the CLF [11], morphological gradient operator [12], nonlinear diffusion [13], and mathematical morphology [14] can also be used in this step.

After the edge map is subtracted, a morphological dilation is applied to generate a continuous edge map. The dilated edge map is then multiplied with the original image, yielding a grayscale edge map. In this way, the pixels on the dilated edge map contain information from the original image and these pixels are used to predict the pixel intensity in the smooth area. 

(*2) Reconstruction.* In the previous section, four major interpolation based reconstruction methods were reviewed. The weighted average (1) method utilizes all the information from the eight neighbors but is sensitive to noise. Unfortunately, noise commonly exists in medical image applications. To be more robust to noise, the weighted median and central weighted median (2) can be used. In this way, only one of the neighbors is used to predict the new pixel value. This replication solves the noise problem but also results in another problem. That is, in some areas with a low gradient change, such as the breast tissue in the mammogram image, this reconstruction may mistakenly yields a large uniform region.

To get a good balance, a weighted alpha trimmed mean can be used in the reconstruction. Each of the eight neighbors is assigned with the weighted intensity *x*_*k*_ = (1/*d*_*k*_)*t*_*k*_, where *t*_*k*_ is the actual edge pixel intensity and *d*_*k*_ is the distance between the pixel to be predicted and edge pixel on a specific direction. Then sort values of all the neighbors in ascending order such that *x*_1_ ≤ *x*_2_ ≤ ⋯≤*x*_*K*_. Let *T*_*α*_ = ⌈*αK*⌉ (the nearest integer greater than or equal to *αK*) be the number of the smallest and largest pixel values to be trimmed or discarded from the sorted sequence, *x*_1_, *x*_2_,…, *x*_*K*_. The alpha trimmed mean [15] is defined by

(8) $$\begin{array}{c} X_{\alpha }=\frac{1}{K-2T_{\alpha }}\overset{K-T_{\alpha }}{\underset{i=T_{\alpha }+1}{\sum }}x_{i}. \end{array}$$

The alpha trimmed mean will be different when the parameter *α* changes. For example, it will be the mean value of the image for *α* = 0 and the median value of the image if *α* is close to 0.5. In this way, the parameters can be tuned for different applications. In this paper, the results are obtained by discarding the maximum and minimum neighbors in the calculation of alpha weighted mean. 

(*3) Similarity Measure NREM.* The reconstructed image is then compared to the original image using a similarity measure, which is then used as an assessment of the edge map. The SSIM [10] is widely used in clean images, but it is noted that the performance of the SSIM index degraded substantially when assessing Gaussian blurred images. The noise in medical images is very prevalent and difficult to model. In the experiments, the SSIM does not perform well when the images are subjected to other distortions such as low contrast and blurring effects. To measure the similarity between the original image and the reconstructed image, the GSSIM [16, 17] is used. GSSIM suggests that the gradients of the images to be compared be integrated into the image similarity assessment to penalize dissimilarity in image contours and edges. Thus, the GSSIM index makes comparisons between both *x* and *y* and the gradients of *x* and *y*. The gradients of *x* and *y* specifically indicate the similarity between edges. In the expression of GSSIM, the contrast and structure terms of SSIM are modified as in (9), where *σ*_*x*′_ and *σ*_*y*′_ represent the standard deviation of the gradient magnitude of *x* and *y*, respectively:

(9) c(x′,y′)=2σx′σy′+C2σx′2+σy′2+C2,s(x′,y′)=σx′y′+C3σx′σy′+C3.

Using similar methods to fuse luminance, gradient contrast, and gradient structure together, the GSSIM over subblocks can be shown in the following:

(10) $$\begin{array}{c} \text{GSSIM}\left(x,y\right)={\left[l\left(x,y\right)\right]}^{\alpha }{\left[c\left(x^{\prime },y^{\prime }\right)\right]}^{\beta }{\left[s\left(x^{\prime },y^{\prime }\right)\right]}^{\gamma }. \end{array}$$

Therefore, the mean of GSSIM over the entire image can be used to indicate the similarity between the reconstructed image and the original image:

(11) $$\begin{array}{c} \text{MGSSIM}\left(x,y\right)=\frac{1}{N}\overset{N}{\underset{i=1}{\sum }}\text{GSSIM}\left(x_{i},y_{i}\right). \end{array}$$

It is worth noting that the similarity measure itself is not accurate enough to measure the reconstruction performance. The reason is that when more edge pixels exist in an edge map, more information from the original image is used in the reconstruction which will definitely yield more similar results. Therefore, a penalty factor *f*_*p*_ which is formulated as a decreasing function of the total number of edge pixels is introduced in (12), where *N*_*e*_ represents the total number of edge pixels in the dilated edge map and *MN* represents the total number of pixels in the original image:

(12) $$\begin{array}{c} f_{p}=\frac{1}{1+N_{e}/MN}. \end{array}$$

The final nonreference based edge measure (NREM) is comprised as the alpha weighted product of these two terms. In this paper, the results are shown with *α* = 1 and *β* = 3, which were obtained experimentally:

(13) $$\begin{array}{c} \text{NREM}\left(i,e\right)={\left(\text{MGSSIM}\right)}^{\alpha }f_{p}^{\beta }. \end{array}$$

## 4. Experimental Results

Edge detection plays an important role in medical image processing as it determines the structure of objects in images. In this section, we demonstrate some applications of the new nonreference edge measures NREM on medical image processing. The testing images are obtained from the Frederick National Library for Cancer Research Database [18].

**Figure 2:**
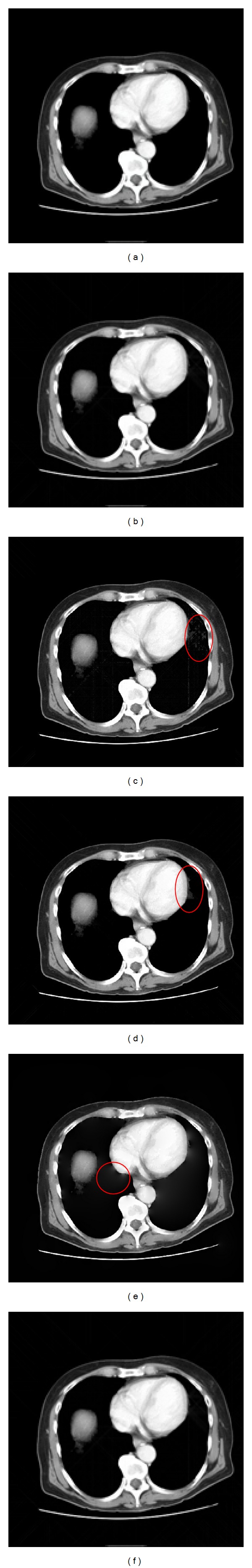
Reconstruction results using different interpolation methods. (a) Original CT kidney image. (b)–(f) Reconstructed results from (b) weighted average, (c) weighted median, (d) central weighted median, (e) PDE, and (f) alpha trimmed weighted average.

**Figure 3:**
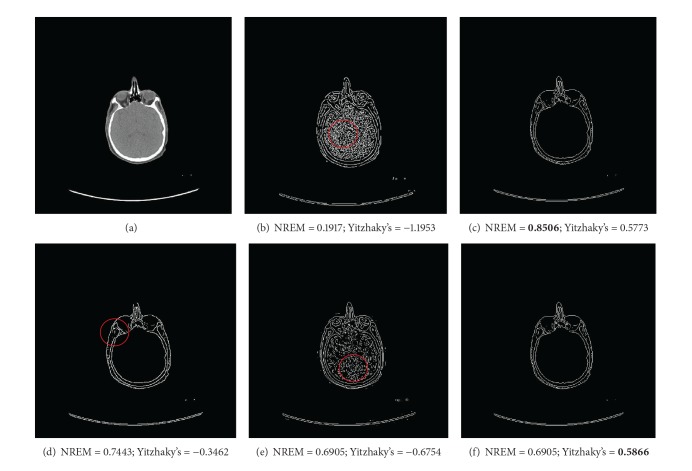
Using measures to select optimal edge detection algorithms. (a) Original CT abdomen image. (b)–(f) Edge detection results from (b) Canny, (c) Sobel, (d) Roberts, (e) LoG, and(f) Prewitt. The optimal edge maps with highest NREM value and Yitzhaky's measure value are in bold.

The first example compares the reconstruction results using the weighted mean, weighted median, central weighted median, PDE, and alpha trimmed weighted mean based interpolation.  Figure 2 shows the original CT kidney image and five reconstructions. It is seen from the results that, for the low quality medical images, the mean based reconstruction is not as sharp as the median based reconstruction. However, the median based reconstruction introduces some artificial lines. As analyzed before, the alpha trimmed mean can be converted to mean or median filter with different parameter alpha. In Figure 3, the alpha trimmed weighted mean reconstruction achieves a good balance. The central weighted median retrieves details but also introduces false details especially around the edge of the real tissues. The PDE based painting method suffers from severe blurring effects.

The second example is using the nonreference edge measures selecting the optimal edge detection algorithm. In Figure 3, multiple edge detection results from the Canny, Sobel, Roberts, Log, and Prewitt for a CT abdomen image are shown in Figures 3(b)–3(f). These edge detection algorithms shown in Figure 3 are commonly used edge detection algorithms and each has its advantages and disadvantages. For example, gradient based edge detection algorithms such as Sobel and Prewitt are simple but sensitive to noise. The Canny edge detector improves the signal to noise ratio by smoothing the image; however, the smoothing may lead to loss of corners and detection of double edges. Therefore, it is necessary to have a reliable edge measure that can help to decide the optimal edge detection algorithm for a specific image. The NREM selects the Sobel edge detection result as the optimal and Yitzhaky's method selects the Prewitt. These two edge detection results agree with the visual assessment. As a comparison, the Canny and LoG edge subtract all the soft tissues inside the abdomen, while the Roberts edge has the disconnection problem on the key edges.

**Figure 4:**
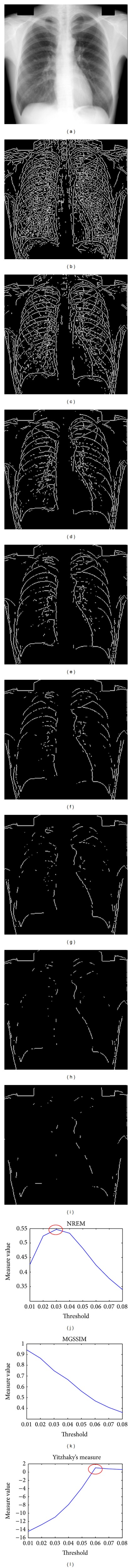
Applying edge measures in assisting in selecting optimal operating parameters. (a) Original X-ray chest image. (b)–(i) Edge detection results using the Sobel edge detector with the threshold ranging from 0.01 to 0.08. (j) Presented measure plot indicating *T* = 0.03 as the optimal parameter value. (k) Measure plot of using MGSSIM alone. (l) Yitzhaky's method indicating *T* = 0.06 as the optimal parameter value.

Another example of using the edge measure as a means of selecting optimal parameter values is shown in Figure 4. In this experiment, the Sobel edge detection algorithm with different threshold ranging from 0.01 to 0.08 is used. The testing image is an X-Ray chest image which suffers from low contrast. Therefore, lower threshold values tend to keep more soft tissue or other noise components in the edge map, while higher threshold values discard essential edges. The proposed measure selects the optimal parameter at threshold = 0.03 and achieves the best tradeoff between noise removal and feature extraction. In contrast, Yitzhaky's method selects threshold = 0.06 which losses some ribs in the edge map.  Figure 4(k) also illustrates the need for the edge pixel density function in the formulation of NREM as the use of MGSSIM alone results in the discussed edge pixel bias.

## 5. Conclusions

Nonreference edge measure is very useful in medical image segmentation, registration, and reconstruction. A new nonreference edge map evaluation NREM for medical applications is proposed in this paper. This measure is based on the fact that the best edge map results consist of the least number of edge pixels at their correct locations needed to characterize all the relevant structures in the reconstruction image. Comparison with state-of-the-art nonreference edge detection measure shows the advantages of the new measure: the NREM utilizes the information from the original image and thus can achieve better performance. Experimental results on using the NREM on selecting the optimal edge detection algorithm and optimal operating parameters show that the measure coincides with subjective evaluation, validating the usefulness of the measure.

## Figures and Tables

**Table 1:** Basic steps in Yitzhaky's NR edge measure.

Yitzhaky's edge measure	
(1) Generate *N* edge detection results *D*_*i*_, *i* = 1,…, *N* using *N* combinations of parameters	
(2) Generate *N* potential ground truth PGT_*i*_, *i* = 1,…, *N*	
(3) Calculate the average true positive (TP), true negative (TN), false positive (FP), and false negative (FN) rate for each potential ground truth	
(4) Construct the correspondence threshold ROC curve (CT-ROC)	
(5) Extract the estimated ground truth using either a diagnosis line or the chi-square estimation	
(6) Select the best edge map which gives the best match to the estimated ground truth	
